# Character strengths of women with polycystic ovary syndrome in a single center

**DOI:** 10.1371/journal.pone.0266398

**Published:** 2022-04-01

**Authors:** Ghina Ghazeeri, Nour Ibrahim, Fatin Khalifeh, Christine Beyrouthy, Lina El-Taha, Maya Bizri

**Affiliations:** 1 Department of Obstetrics and Gynecology, Faculty of Medicine, American University of Beirut Medical Center, Beirut, Lebanon; 2 Department of Psychiatry, Faculty of Medicine, American University of Beirut Medical Center, Beirut, Lebanon; Lewis Katz School of Medicine at Temple University, UNITED STATES

## Abstract

**Purpose:**

To investigate the difference in character strengths (CSs) between patients with and without polycystic ovary syndrome (PCOS) and the association between biological (i.e., testosterone levels) and psychological factors (i.e., character strengths).

**Patients and methods:**

A total of 99 women divided into PCOS (49) and non-PCOS (50) groups who presented to the gynecological clinics at the women’s center in the American University of Beirut Medical Center in 2017 were included. Women were assessed for testosterone bioavailable levels and completed a questionnaire that included Hospital Anxiety and Depression Scale and Values in Action Survey-72. Univariate and multivariable analyses were performed to examine the association of CSs between the two groups and its predictors.

**Results:**

The scores of hope, judgement, perspective, and transcendence of the PCOS group were significantly higher in comparison with healthy participants. An increase in free androgen index was negatively correlated to the score of judgement only.

**Conclusion:**

Women with PCOS have their own profile of character strengths and virtues that constitute judgement, hope, perspective, and transcendence. This in turn can be utilized to reinforce those personality strengths and thus decrease the psychological distress and mood disorders accompanied with this disease.

## Introduction

Polycystic ovarian syndrome (PCOS) is a hyperandrogenic heterogeneous endocrine disorder that affects 15–20% of women in their reproductive age when using Rotterdam criteria [1]. It is characterized by androgen excess, ovulatory dysfunction, and/or polycystic ovaries which have psychological, economic, and social consequences [2, 3]. Further, it is a chronic condition associated with a variety of clinical sequalae that may have a lifespan impact on PCOS patients. Such features include reproductive problems (hirsutism, androgenic alopecia, obstetric complications, and infertility), metabolic manifestations (obesity and insulin resistance), and psychological implications (emotional distress, depression, anxiety, and sexual arousal impairment) [4–6]. Interest in psychological aspects of PCOS patients has grown as concomitant symptoms are being increasingly recognized by practicing clinicians. In one cohort study, Hung and colleagues (2014) postulated that PCOS patients had increased odds of depressive, anxiety, and sleep disorders [7].

PCOS is characterized by chronically augmented androgens, specifically free testosterone levels [8]. Testosterone is a crucial steroid hormone involved in various biological processes including reproductive function, brain development, and behavioral traits [9]. It is one of the most studied hormones in relation with human behavior. Indeed, previous studies have reported the interrelation between testosterone levels and personality variations [10, 11]. Some studies have demonstrated the association of higher levels of testosterone with certain traits such as extraversion, higher sensation seeking, and low-risk aversion [12–14].

Recently, the role of character strengths (CSs), within the field of positive psychology, has gained increasing interest and attention in patients dealing with chronic illnesses [15]. As a concept, character strengths are considered positive traits reflected in thoughts, feelings, and behaviors. They contribute to one self’s fulfillment, happiness, and life satisfaction [16]. In 2004, a classification of 24 identified character strengths, allocated to one of the 6 universal virtues, was proposed by Peterson and Seligman [17]. Numerous reports have emphasized the benefit of character strengths use in enhancing psychological and physiological well-being [18]. In fact, several studies and reviews revealed the positive effects of character strength-based intervention in patients with chronic illnesses [15]; however, none have explored whether any differences of character strengths exist between patients with and without PCOS. Therefore, this study aimed to investigate the difference in CSs between PCOS and non-PCOS women and whether it is associated with biological predictors (testosterone levels).

## Materials and methods

### Design

This was a descriptive study performed using a cross-sectional research design of 99 participants in 2017. It was conducted in the gynecological clinics at the women’s center at the American University of Beirut Medical Center (AUBMC). The study was approved by the Institutional Review Board (IRB) of the American University of Beirut (AUB) (SBS-2017-0491). Written informed consent form was obtained from each participant. The study with its objectives and procedures were fully explained and discussed with each subject before administering the questionnaires.

The 99 participants were divided into two groups: PCOS (49) and non-PCOS (50). For the PCOS group, participants were eligible for this study if they met the following requirements: women of child-bearing age (18–45 years old) who met 2 out of 3 criteria of the Revised Rotterdam Consensus for PCOS (oligo- or anovulation; clinical and/or biochemical signs of hyperandrogenism; polycystic ovaries). All controls included women with normal fertility or subfertility due to tubal, male factor or unexplained cause, who did not fit the Rotterdam criteria and were not on contraceptive or anti-androgen pills. Exclusion criteria for the PCOS group included endocrine disorders such as congenital adrenal hyperplasia, Cushing’s syndrome, menopause, androgen secreting tumors, thyroid dysfunction, hyperprolactinemia, type 2 diabetes mellitus, impaired glucose tolerance, concomitant cardiovascular disease, liver and renal dysfunction, and the use of any medication that interferes with endocrine and psychiatric parameters. Besides, patients with any physical disability, clinically proven psychiatric disease, or cut-off score of Hospital Anxiety and Depression Scale (HADS), assessed by a psychiatrist, were excluded.

Body mass index (BMI) was calculated as body weight divided by the square of the body height (kg/m^2^). To measure biological hyperandrogenism, venous blood sampling for testosterone bioavailable levels [testosterone and sex hormone-binding globulin (SHBG)] were taken. For clinical assessment of hyperandrogenism, hirsutism score was calculated according to Ferriman and Gallwey (1961) scoring system. The participants were evaluated according to body hair on 9 regions from scale 0 (no hair) to 4 (frankly virile), and these were summed to provide an overall hirsutism score. The result was considered either normal (total score less than 8), mild (total score of 8 to 15), or moderate to severe hirsutism (total score greater than 15). Moreover, pelvic ultrasound was administered by the primary physician to provide a report of polycystic ovaries.

### Measures

Participants were asked to complete a data collection sheet that consisted of sociodemographic questionnaire, HADS and Values in Action (VIA) Survey-72:

The sociodemographic data included: age, weight, height, socioeconomic class (income), employment, education, marital status, number of children, smoking status, alcohol consumption, infertility, hirsutism, and personal and family medical history.

The HADS was used to assess psychological distress. It is a reliable and valid self-reported questionnaire consisting of 14 questions in a 4-point-likert scale which assesses the overall severity of anxiety and depression (7 questions each). The cut off points are 10 and 7 for depression and anxiety, respectively. Participants who scored high on HADS were informed by their primary physician regarding any significant results.

The VIA Survey-72 (S1 Table), a shorter version of the VIA Survey-240, consisting of 72 questions was used to assess 24 character strengths. For each question, a five-point scale was used ranging from 1 (very much unlike to me) to 5 (very much like me).

### Statistical analyses

Data was analyzed using SPSS 25 statistical software package (IBM, USA). Descriptive statistics were used to report the sample demographic characteristics. T-test analyses were performed to examine the association of the character strengths and virtues between the two groups. Moreover, multiple linear regression analyses were used to assess the predictors and their contributions to the scores of character strengths and virtues. The significance was set at 0.05.

## Results

**Fig 1:**
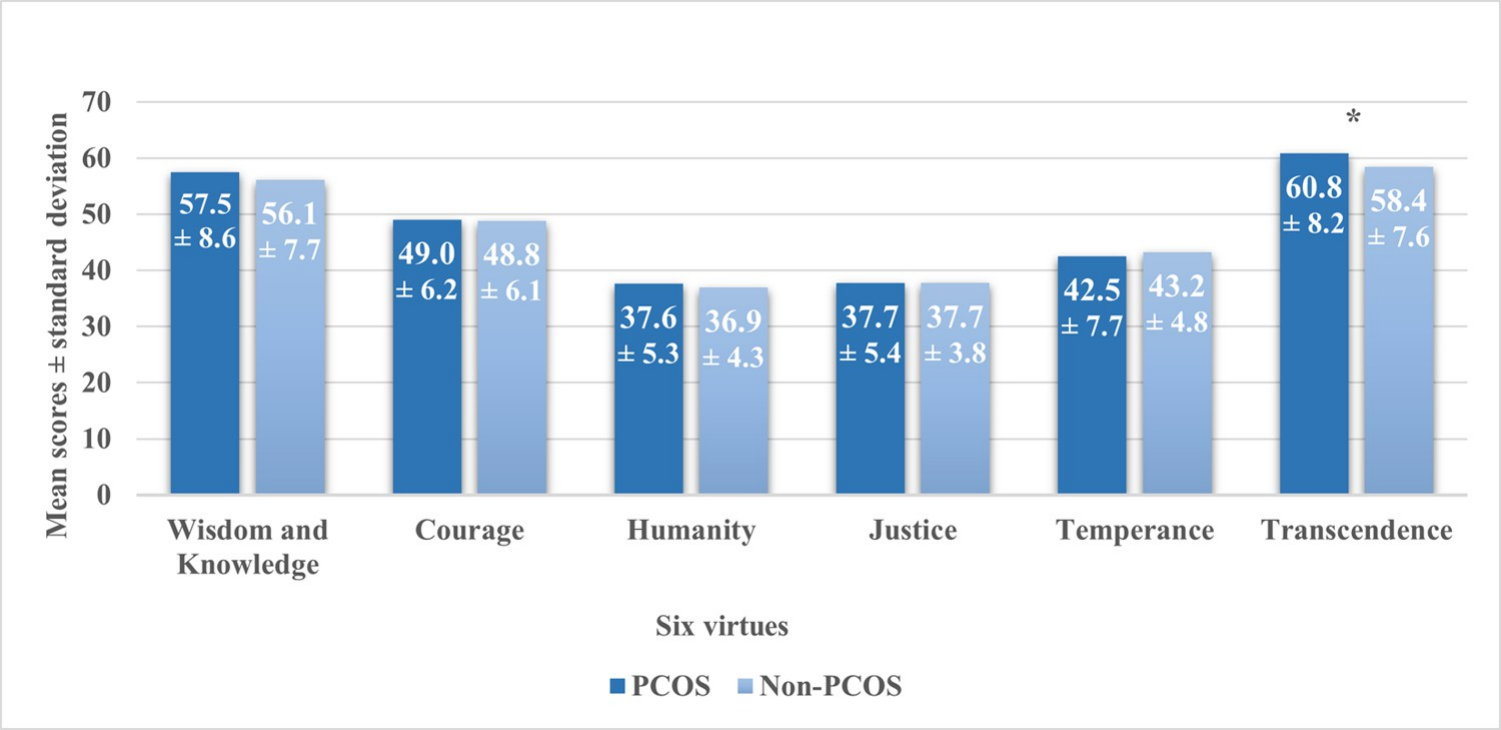
Comparison of 6 virtues between the two groups [PCOS (n = 49); non-PCOS (n = 50)]. *: indicates significance (p-value < 0.05).

Among the 99 female participants, there were 49 women with PCOS and 50 without. The age of non-PCOS women had a mean ± SD of 28.1 ± 5.3 years, while that of PCOS women was 25.0 ± 4.7 years. Regarding their educational level, the majority (63.3%) of PCOS group had a bachelor’s degree (or higher) which was lower than that of non-PCOS group (82.0%). As for employment, only 51.0% of PCOS participants were employed which is much lower in comparison to that of non-PCOS participants (90.0%). Moreover, most of PCOS and non-PCOS groups had a household monthly income between 1,000$ and 2,000$ (35.4% and 43.8%, respectively). The percentage of alcohol intake was lower in PCOS participants (8.0%) than that of non-PCOS (26.0%). In contrast, the smoking history was approximately similar between the two groups. **Table 1** depicts this information. **Table** 2 presents the distribution of anxiety and depression scores between PCOS and non-PCOS women. There were significant differences only in the score of depression between the two groups (p < 0.05). The scores of hope, judgement, and perspective of the PCOS group were significantly higher (p < 0.05) than those of the non-PCOS group **(****Table** 3**)**. In addition to this, t-test results showed that there was a significant difference (p < 0.05) in the transcendence score between the two groups. However, none was present for the other 5 virtues **(Fig 1)**. **Table** 4 shows the predictors’ results of character strengths and virtues for the two studied groups. Age, hirsutism, BMI, bioavailable testosterone, SHBG, free androgen index (FAI), irregular menstrual cycle, acne, depression and anxiety scores, and infertility were the independent variables used to fit several regression models. Only the fitted regression models with significant results with respect to character strengths and virtues are displayed in **Table** 4. The results of linear regression showed that an increase in FAI tends to decrease the score of judgement. Also, an increase in acne or hirsutism score leads to a decrease in the score of appreciation of beauty and excellence in PCOS participants. Moreover, irregularity of menstrual cycles causes an increase in the scores of transcendence and curiosity.

**Table 1:** Distribution of PCOS and non-PCOS groups with respect to their individual characteristics.

Variables		PCOS	Non-PCOS
		(n = 49)	(n = 50)
		N (%) or Mean (SD)	N (%) or Mean (SD)
**Age**		25.0 (4.7)	28.1 (5.3)
**Education**	Less than high school	5 (10.2)	0 (0.0)
	High school	3 (6.1)	3 (6.0)
	College degree	10 (20.4)	6 (12.0)
	Bachelor’s degree or higher	31 (63.3)	41 (82.0)
**Employment**	Unemployed	24 (49.0)	5 (10.0)
	Employed	25 (51.0)	45 (90.0)
**Household monthly income (x)**	x < 1000$	12 (25.0)	9 (18.7)
	1,000$ < x < 2,000$	17 (35.4)	21 (43.8)
	2,000$ < x < 5000$	14 (29.2)	16 (33.3)
	5,000$ < x < 10,000$	4 (8.3)	2 (4.2)
	x > 10,000$	1 (2.1)	0 (0.0)
**Alcohol**	No	45 (92.0)	37 (74.0)
	Yes	4 (8.0)	13 (26.0)
**Smoking**	No	34 (69.0)	37 (74.0)
	Yes	15 (31.0)	13 (26.0)

Note: valid percentages were used in case of no response.

**Table 2:** Comparison of anxiety and depression scores between PCOS and non-PCOS groups.

Variables	PCOS (n = 49); Mean (SD)	Non-PCOS (n = 50); Mean (SD)	t	P-value
**Anxiety Score**	5.5 (4.0)	5.9 (3.7)	0.52	0.60
**Depression Score**	3.3 (2.7)	4.7 (3.4)	2.21	0.03

**Table 3:** Comparison of 24 character strengths between the two groups.

Variables	PCOS (n = 49); Mean (SD)	Non-PCOS (n = 50); Mean (SD)	P-value
**Appreciation of Beauty and Excellence**	12.6 (1.8)	12.1 (1.7)	0.088
**Bravery**	12.0 (2.1)	12.0 (2.5)	0.571
**Creativity**	11.8 (2.4)	11.4 (2.2)	0.297
**Curiosity**	11.6 (2.0)	11.2 (2.0)	0.362
**Fairness**	12.3 (2.2)	12.42 (1.8)	0.843
**Forgiveness**	11.7 (2.7)	11.3 (2.3)	0.184
**Gratitude**	12.2 (2.4)	12.0 (2.0)	0.310
**Honesty**	13.2 (1.6)	13.0 (1.6)	0.495
**Hope**	12.3 (2.2)	11.6 (2.1)	**0.037**
**Humility**	9.9 (2.5)	10.6 (2.0)	0.341
**Humor**	12.0 (2.7)	11.68 (2.2)	0.276
**Judgement**	12.5 (2.2)	12.1 (1.7)	**0.045**
**Kindness**	12.6 (1.8)	12.5 (1.8)	0.680
**Leadership**	12.8 (1.6)	12.3 (1.7)	0.227
**Love**	12.7 (2.6)	12.2 (2.3)	0.153
**Love of learning**	9.5 (2.6)	9.8 (2.1)	0.540
**Perseverance**	12.3 (2.1)	12.5 (2.0)	0.532
**Perspective**	12.1 (1.9)	11.6 (1.9)	**0.043**
**Prudence**	11.6 (2.8)	11.4 (2.2)	0.362
**Self-regulation**	9.4 (2.6)	9.9 (2.5)	0.367
**Social intelligence**	12.4 (1.7)	12.1 (1.6)	0.326
**Spirituality**	11.6 (2.5)	11.0 (2.4)	0.206
**Teamwork**	12.6 (2.5)	13.0 (1.2)	1.000
**Zest**	11.5 (2.7)	11.2 (2.5)	0.221

**Table 4:** Multiple linear regressions for the predictors of the 24 character strengths and 6 virtues between the two groups.

Dependent Variable	Variables	B	Beta	t	P-value	R^2^
**Appreciation of Beauty and Excellence**	Acne	-1.333	-0.326	-2.308	0.024	0.144
	Hirsutism	-0.073	-0.240	-2.005	0.049	
**Curiosity**	Irregular menstrual cycle	0.930	0.244	2.244	0.028	0.188
**Judgement**	Free androgen index (FAI)	-0.093	-0.227	-1.994	0.05	0.101
**Transcendence**	Irregular menstrual cycle	3.062	0.232	2.013	0.048	0.54

## Discussion

To better understand the personality and psychological profile of PCOS women and its association with biological markers, we compared the character strengths between PCOS and non-PCOS groups. We found that higher scores of hope, judgement, perspective, and transcendence were present within the PCOS women sample.

Various previous studies revealed the association of psychopathology in PCOS sample population [19, 20]. However, in the present study, levels of depression and anxiety were in the normal range for both groups, with PCOS women scoring even lower than those of the control group for depression. In fact, participants in PCOS group with a history of mental or psychological disorders were excluded in our study. Their exclusion may have pertained to a lowered difference or association of psychopathology traits in PCOS group, as the latter was not the focus of the present study. Further, in one study, Weiner et al. [21] revealed a weak non-linear relationship between mood and testosterone. However, our results do not echo the latter finding as there was lack of any correlation between the two studied variables.

Testosterone exerts activational and organizational influences on brain structures and its behavior among both sexes [22]. Although multiple studies on rodents and few on humans aimed to study the behavioral traits of both genders in relation to testosterone levels, some results showed either a negative or positive association but none displayed a causal relationship between the two [23]. Thus, the role and activational effects of testosterone on behavior and personality profile of women are still controversial and under debate. In our study, there was no existence of correlations between testosterone levels and character strengths for both PCOS and control groups. This lack of association proposes a little to no activational influences of testosterone on the character strengths of the sample population.

To the best of our knowledge, this was the first study to ever assess the character strengths profile and its predictors in a sample group of PCOS women. We found that, of the 24 CSs, PCOS patients had higher scores of judgement, hope, and perspective than the healthy controls. As for the 6 virtues, only transcendence score was shown to be significantly higher in the PCOS group. According to Peterson and Seligman (2004), judgement is considered a corrective strength such that it counteracts faulty thinking [24]. Possessing such character trait gives the person the ability to examine, evaluate, and weigh evidence from all sides prior to jumping into conclusions. Indeed, judgement is described as a core strength of the mind. Likewise, the character strength of hope facilitates the adoption of an optimistic explanatory approach towards events and action-oriented strength towards the future [17]. Perspective, on the other hand, allows the individual to have an eye on the bigger picture instead of being consumed by all the little irrelevant details [24]. Moreover, the virtue of transcendence aids in the development of a better connection with the outer universe and its meaning [17]. These findings might indicate possible good problem-solving skills and positive reappraisal strategies among PCOS patients. Saei Ghare Naz et al. identified that PCOS women who adopted positive adaptive coping to their disorder had hopefulness as their driving force [25]. However, this work does not come in accordance with a study that revealed higher neuroticism levels in patients with PCOS [26]. It is important to note that this is the first time the VIA survey was used on PCOS patients, and this tool does not have normative values established yet, so the results need to be interpreted with caution until further studies confirm the findings.

In the present study, we performed regression analyses to find out predictors of character strengths’ scores related to the symptoms of their condition. Our findings showed that acne and hirsutism were negatively associated with the appreciation of beauty and excellence score. In fact, Bazarganipour et al. indicated that hirsute PCOS women had poorer body image [27]. FAI was weakly correlated to lower scores of judgement. Contrarily, one study revealed that higher basal testosterone levels had a positive correlation with self-direction and decisiveness among women [10]. Moreover, menstrual cycle disturbances were linked to higher curiosity and transcendence scores.

Our study has some limitations worth noting. Although there was no mean difference between the two studied groups, the sample size was small. Another limitation was the implementation of the project in gynecologic clinics of one hospital. Thus, the PCOS sample might not be an appropriate representation of other PCOS women. Moreover, the present study was the first work to reveal a certain character strengths profile along with its predictors among PCOS women. This calls for population-wide studies to establish normative scores of each of VIA elements, as well as future large, multicenter studies on PCOS women to confirm the findings of our study.

## Conclusion

In conclusion, we showed that women with PCOS have higher scores of judgement, hope, perspective, and transcendence as character strengths in comparison with healthy patients. We suggest that it is essential for PCOS patients to be referred to psychiatric consultations and undergo positive psychology therapy to enhance their character strengths from the onset of diagnosis. This might be a cost-effective, efficient prophylactic means for adapting with the syndrome and preventing psychopathological disorders associated with PCOS women on the long run. Prospective studies in larger groups are warranted to assess this issue.

## Supporting information

S1 Table This table shows Values in Action (VIA) Survey-72. (DOCX)
