# Supplementary material for: Character strengths of women with polycystic ovary syndrome in a single center
Source: PLoS One. 2022 Apr 1;17(4):e0266398. doi: 10.1371/journal.pone.0266398 (PMC8975165; doi:10.1371/journal.pone.0266398)
Supplement: S1 Table — (DOCX) [file pone.0266398.s001.docx]

**S1 Table. This table shows Values in Action (VIA) Survey-72.**

|  |  | | | | | |
| --- | --- | --- | --- | --- | --- | --- |
|  | Rate the following statement starting from “strongly disagree to strongly agree”: | | | | | |
|  |  | Strongly Disagree | Disagree | Neutral | Agree | Strongly Agree |
| 1 | I have taken frequent stands in the face of strong opposition. |  |  |  |  |  |
| 2 | I never quit a task before it is done. |  |  |  |  |  |
| 3 | I always keep my promises. |  |  |  |  |  |
| 4 | I always look on the bright side. |  |  |  |  |  |
| 5 | I am a spiritual person. |  |  |  |  |  |
| 6 | I know how to handle myself in different social situations. |  |  |  |  |  |
| 7 | I always finish what I start. |  |  |  |  |  |
| 8 | I really enjoy doing small favors for friends. |  |  |  |  |  |
| 9 | As a leader, I treat everyone equally well regardless of his or her experience. |  |  |  |  |  |
| 10 | Even when candy or cookies are under my nose, I never overeat. |  |  |  |  |  |
| 11 | I practice my religion. |  |  |  |  |  |
| 12 | I rarely hold a grudge. |  |  |  |  |  |
| 13 | I am always busy with something interesting. |  |  |  |  |  |
| 14 | No matter what the situation, I am able to fit in. |  |  |  |  |  |
| 15 | I go out of my way to cheer up people who appear down. |  |  |  |  |  |
| 16 | One of my strengths is helping a group of people work well together even when they have their differences. |  |  |  |  |  |
| 17 | I am a highly disciplined person. |  |  |  |  |  |
| 18 | I experience deep emotions when I see beautiful things. |  |  |  |  |  |
| 19 | Despite challenges, I always remain hopeful about the future. |  |  |  |  |  |
| 20 | I must stand up for what I believe even if there are negative results. |  |  |  |  |  |
| 21 | I finish things despite obstacles in the way. |  |  |  |  |  |
| 22 | Everyone's rights are equally important to me. |  |  |  |  |  |
| 23 | I see beauty that other people pass by without noticing. |  |  |  |  |  |
| 24 | I never brag about my accomplishments. |  |  |  |  |  |
| 25 | I am excited by many different activities. |  |  |  |  |  |
| 26 | I am a true life-long learner. |  |  |  |  |  |
| 27 | I am always coming up with new ways to do things. |  |  |  |  |  |
| 28 | People describe me as "wise beyond my years." |  |  |  |  |  |
| 29 | My promises can be trusted. |  |  |  |  |  |
| 30 | I give everyone a chance. |  |  |  |  |  |
| 31 | To be an effective leader, I treat everyone the same. |  |  |  |  |  |
| 32 | I am an extremely grateful person. |  |  |  |  |  |
| 33 | I try to add some humor to whatever I do. |  |  |  |  |  |
| 34 | I look forward to each new day. |  |  |  |  |  |
| 35 | I believe it is best to forgive and forget. |  |  |  |  |  |
| 36 | My friends say that I have lots of new and different ideas. |  |  |  |  |  |
| 37 | I always stand up for my beliefs. |  |  |  |  |  |
| 38 | I am true to my own values. |  |  |  |  |  |
| 39 | I always feel the presence of love in my life. |  |  |  |  |  |
| 40 | I can always stay on a diet. |  |  |  |  |  |
| 41 | I think through the consequences every time before I act. |  |  |  |  |  |
| 42 | I am always aware of the natural beauty in the environment. |  |  |  |  |  |
| 43 | My faith makes me who I am. |  |  |  |  |  |
| 44 | I have lots of energy. |  |  |  |  |  |
| 45 | I can find something of interest in any situation. |  |  |  |  |  |
| 46 | I read all of the time. |  |  |  |  |  |
| 47 | Thinking things through is part of who I am. |  |  |  |  |  |
| 48 | I am an original thinker. |  |  |  |  |  |
| 49 | I have a mature view on life. |  |  |  |  |  |
| 50 | I can express love to someone else. |  |  |  |  |  |
| 51 | Without exception, I support my teammates or fellow group members. |  |  |  |  |  |
| 52 | I feel thankful for what I have received in life. |  |  |  |  |  |
| 53 | I know that I will succeed with the goals I set for myself. |  |  |  |  |  |
| 54 | I rarely call attention to myself. |  |  |  |  |  |
| 55 | I have a great sense of humor. |  |  |  |  |  |
| 56 | I always weigh the pro's and con's. |  |  |  |  |  |
| 57 | I enjoy being kind to others. |  |  |  |  |  |
| 58 | I can accept love from others. |  |  |  |  |  |
| 59 | Even if I disagree with them, I always respect the leaders of my group. |  |  |  |  |  |
| 60 | I am a very careful person. |  |  |  |  |  |
| 61 | I have been told that modesty is one of my most notable characteristics. |  |  |  |  |  |
| 62 | I am usually willing to give someone another chance. |  |  |  |  |  |
| 63 | I read a huge variety of books. |  |  |  |  |  |
| 64 | I try to have good reasons for my important decisions. |  |  |  |  |  |
| 65 | I always know what to say to make people feel good. |  |  |  |  |  |
| 66 | It is important to me to respect decisions made by my group. |  |  |  |  |  |
| 67 | I always make careful choices. |  |  |  |  |  |
| 68 | I feel a profound sense of appreciation every day. |  |  |  |  |  |
| 69 | I awaken with a sense of excitement about the day's possibilities. |  |  |  |  |  |
| 70 | Others consider me to be a wise person. |  |  |  |  |  |
| 71 | I believe that it is worth listening to everyone's opinions. |  |  |  |  |  |
| 72 | I am known for my good sense of humor. |  |  |  |  |  |
